# Graphenic Materials for Biomedical Applications

**DOI:** 10.3390/nano9121758

**Published:** 2019-12-11

**Authors:** Daniela Plachá, Josef Jampilek

**Affiliations:** 1 Nanotechnology Centre, VŠB-Technical University of Ostrava, 17. listopadu 2172/15, 708 00 Ostrava-Poruba, Czech Republic; 2 ENET Centre, VŠB-Technical University of Ostrava, 17. listopadu 2172/15, 708 00 Ostrava-Poruba, Czech Republic; 3 Department of Analytical Chemistry, Faculty of Natural Sciences, Comenius University, Ilkovicova 6, 842 15 Bratislava, Slovakia; josef.jampilek@gmail.com

**Keywords:** graphene, graphene oxide, graphene-based nanomaterials, nanoformulations, drug delivery systems, biomedical applications

## Abstract

Graphene-based nanomaterials have been intensively studied for their properties, modifications, and application potential. Biomedical applications are one of the main directions of research in this field. This review summarizes the research results which were obtained in the last two years (2017–2019), especially those related to drug/gene/protein delivery systems and materials with antimicrobial properties. Due to the large number of studies in the area of carbon nanomaterials, attention here is focused only on 2D structures, i.e. graphene, graphene oxide, and reduced graphene oxide.

## 1. Introduction

Nanomaterials are known for their outstanding physicochemical and biological properties, which make them very desirable in material science for different applications. Graphene-based materials (GBMs) that mainly contain zero-dimensional (0D) fullerenes, one-dimensional (1D) carbon nanotubes (CNTs), two-dimensional (2D) graphene (GR), graphene oxide (GO), and reduced graphene oxide (rGO), as well as their doped and functionalized derivatives, are among the most promising materials here. GR is the basic building unit of this large group of materials, including graphite, in which the individual GR layers are assembled into a three-dimensional (3D) structure [1,2]. GBMs are well-known to have extraordinary electrical, optical, thermal, and mechanical properties, which makes them suitable materials for applications in supercapacitors, ultrasensitive sensors, rapid charging/discharging batteries, quantum physics, catalysts, advanced environmental photocatalysts, superadsorbents, nanocomposites (NCs), chemical solar and fuel cells, optoelectronics, electronics, and others [3].

Due to their properties, GBMs provide possibilities for many applications in the biomedical field, such as use in drug, gene, and protein delivery, photothermal therapy, as a material for building biosensors and bioimaging, as potential antimicrobial agents, and as a scaffold for tissue engineering and cell cultures [4,5]. Among all the named materials, 2D carbon nanostructures provide a high specific surface area that allows for the establishment of suitable cell-substance interactions and excellent loading efficiency due to multiple attachment spots, enabling interactions with small drug molecules or biomolecules on the GR material surface. For this reason, GR offers better use in this area than CNTs. Many other theranostic agents based on metal nanoparticles (NPs) and polymers have been proposed in the biomedical field, however, the GR-based systems seem to have better stability in the physiological environment and seem to be more biologically acceptable [6,7,8].

Pure GR is a strongly hydrophobic material and requires surfaces modifications or stabilizing agents in order to be dispersible in water. For that reason, GO and rGO are often used to improve material properties [2]. Both materials contain functional groups on their surfaces which improve their hydrophilic properties, and they can be further functionalized to tailor their properties for intended purposes. On the other hand, the lipophilicity of GR can be used in membrane barrier penetration systems [9].

This review focuses on recent advances in 2D GBM biomedical applications from the past two years. It summarizes general facts about GBMs and their applications in this area. This summary examines drug delivery systems (DDSs) in detail. Other areas of biomedical research are mentioned only marginally, as research on GR and its derivatives is unstoppably fast, moving forward in each area, where each area requires its own attention to specific detailed processing.

## 2. Graphene-Based Materials

**Figure 1:**
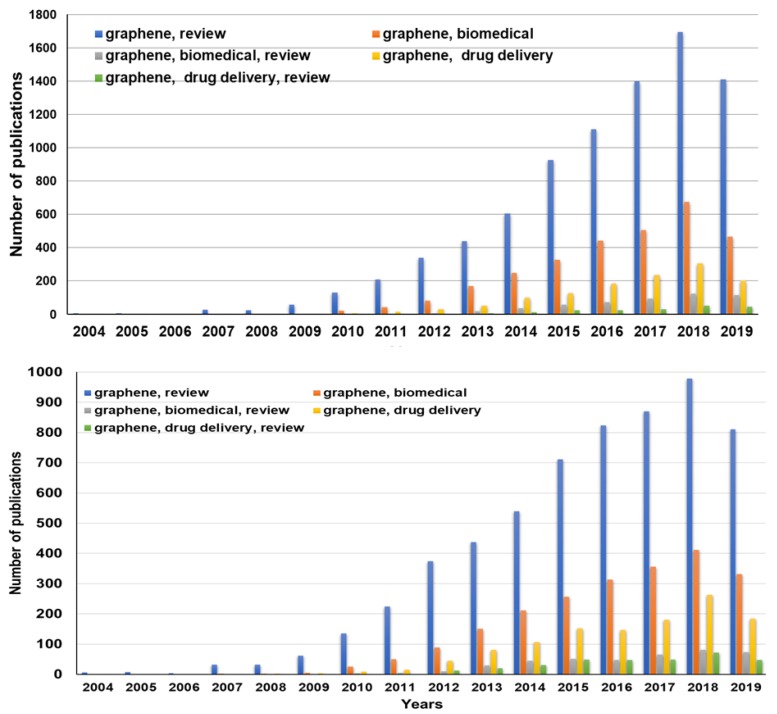
Graphene research: Web of Science (upper part)/Scopus (lower part) review. Data obtained 22 September 2019.

GBMs have been very extensively studied in last few decades. Figure 1 shows the number of publications in the period of 2004–2019, dealing with GR, GR in biomedical applications, and GR in DDSs, taking into account review articles as well. According to Web of Sciences/Scopus, more than 195,900 and 144,700 research articles dealing with GR have been published in this period, respectively (keyword “graphene”). It can be seen from the Figure 1 that GR research in general and in biomedical applications started to increase rapidly after 2010. There is an expectation that research will continue to evolve because of the outstanding properties of GR and derived materials. A growing trend is also evident for research into GR applications in biomedical areas and, for example, in the application of GR for DDSs (keywords “GR, biomedical”; “GR, biomedical, review”; “GR, drug delivery and GR, drug delivery, review”).

**Figure 2:**
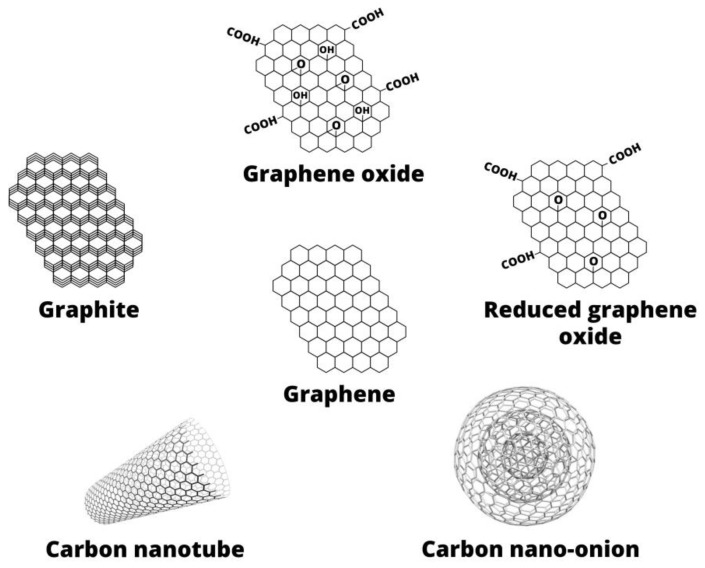
Schematic image of selected graphene-based nanomaterials.

The terms GBMs or graphene family nanomaterial (GFNs) includes GR and its derivatives, such as GO, rGO, GR/GO dots (GD/GOD), GR nano-onions, and GR nano-ribbons, as well as fullerenes, and CNTs, see Figure 2 [10,11].

GR forms a basic structure of GBMs [12]. It is a 2D-planar sheet/crystal of one-atom thickness, consisting of sp^2^ hybridized carbon atoms. The carbon atoms are arranged into a honeycomb lattice, in which each carbon atom is bound to three adjoining carbon atoms via two single and one double covalent bond [13,14,15]. GR can exist in a single layer, few-layered structure, or forms a graphitic structure. GR is well-known for its exceptional and outstanding mechanical properties, such as an exceptional structural rigidity, accompanied a fracture strength of 130 GPa, an elastic modulus of 32 GPa, and a 1 TPa Young’s modulus. It exhibits high electrical conductivity (10^4^ S/cm), ultra-high thermal conductivity (5300 W/mK), and excellent electron mobility (250,000 cm^2^/Vs) at room temperature, which is caused by a presence of a delocalized π-electron system in the carbon lattice, where each carbon atom contributes to this system by one unbound electron freely moving through the crystal. The other significant properties are a large specific surface area of 2630 m^2^/g, the unusual carrier transport velocity which reaches up to 40 GHz, the charge carrier concentration of 1.4 × 10^13^ cm^−2^, and high-current carrying capacity of up to 10^9^ A/cm^2^. Optical transmittance of 97.7% has confirmed these superior optical properties [14,16,17,18].

One can imagine GR as a polycyclic aromatic macromolecule which forms a planar structure with a large surface area, allowing the adsorption and anchoring of many compounds, such as biomolecules, metals, and fluorescent species. The compounds can interact with the system of delocalized π-electrons via electrostatic interactions and non-covalent π-π stacking. These kinds of interactions are one of the processes of GR surface functionalization [12,19,20].

**Figure 3:**
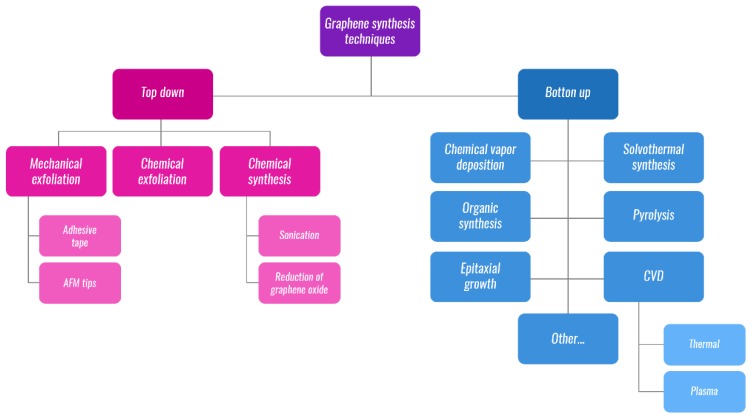
Selected methods of graphene synthesis.

There are several procedures for the synthesis of GR, and two major methodological approaches can be distinguished, namely, the ‘bottom-up’ and ‘top-down’ approaches (Figure 3). There are two main considerations for GR fabrication, namely, producing GR of high quality and simultaneously producing said GR in a high quantity [5,14]. Several methods have been properly investigated, such as the mechanical or chemical exfoliation of graphite, the epitaxial growth of GR on carbides of silicon, tantalum, or titanium, or on different metal substrates, such as nickel, copper, cobalt, iridium, platinum, etc. Other methods, such as chemical vapor deposition, solvothermal or organic synthesis could be also named, or synthesis of GR from graphite oxide through oxidation of graphite eventually via un-zipping of CNTs [14,19,21].

GO is derivative of GR. It is a flake-like material that is characterized by the occurrence of polar oxygen-containing functional groups. The GO flakes consist of a typical graphenic planar structure, composed of sp^2^ carbon atoms that is disrupted by the presence of carbon atoms in the sp^3^ hybridization state. While on the planar surface, there are mainly hydroxyl and epoxy functional groups, while at the edges of the flakes there are carboxylic groups [18,22,23]. This composition is a reason for the aforementioned hydrophilicity, the good aqueous dispersity, and the excellent ability to easily cross cell membranes. The functional groups also allow for the capture, anchoring, or immobilization of polymers or biomolecules, such as nucleic acids, as well as drugs, NPs, etc., [24] and, for that reason, GO is a suitable carrier for delivering proteins, peptides, RNA, DNA, and various molecules of biologically active substances with antiviral, antibacterial, or anticancer activities into cells [25].

**Figure 4:**
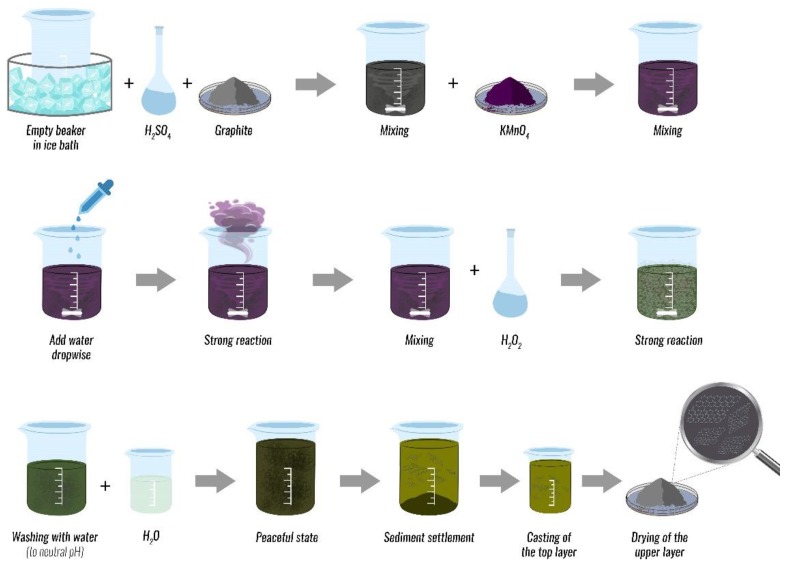
Scheme of graphene oxide (GO) preparation using Hummers’ method.

For applications in the biomedical area, especially for delivering systems or antimicrobial materials, GO is usually synthetized from graphite using various modifications of Hummers’ method. One possibilities for a modified Hummers’ method is schematically shown in Figure 4. The effective delamination of graphite can be achieved in at a high yield by selecting suitable reagents and conditions for the manufacturing process. Thus, it can be produced in large quantities at affordable costs. The fully delaminated material may have a thickness of about 1 nm [16,19,21]. The polar groups are usually further modified by functionalization with the biocompatible and hydrophilic polymers, such as polyethylene glycole (PEG) or polyvinylpyrrolidone (PVP), then different drugs are loaded on the GR plane. Among GBMs, GO is an excellent material for DDSs, precisely because the presence of functional groups makes it easy to modify its surface [12,26].

The interactions of GO flakes are strongly influenced by their size. Functionalized GO flakes can be easily broken down to a smaller size by mild sonication and then are able to enter into cells. It is known that GOs with lateral dimensions higher than 100 nm do not easily penetrate into cells, however GO flakes of approximately 100 nm and less can readily cross the cell membrane. If the diameter reaches below 40 nm, their penetration in the cells may be further increased [27].

The reduction of GO functional groups results in rGO. The structure of rGO is similar to that of GR, and it is associated with its higher electrical conductivity compared to GO [5,22,28]. Simultaneously, due to the lower content of oxygen-containing functional groups, it is more hydrophobic. Large-scale rGO production is usually performed in two steps. First, the graphite is chemically oxidized to form GO, which is then reduced to rGO. Hydrazine is the best-known reductant here, however, it is poisonous and hazardous for the environment. Due to this fact, alternatives have been studied, such as organic acids, amino acids, proteins, sugars, microorganisms, plant extracts, and antioxidants, which are called “green reducing agents” [29]. Another method of reduction is thermal, solothermal, electrochemical, microwave, and photoreduction [11,19,23]. It was found in some instances that not all functional groups were eliminated during the reduction, and some of them remained preserved on the rGO basal plane, e.g., –OH, –C–O–C–, –CO, and –COOH. The resulting properties of rGO are influenced by the reduction method used and the conditions under which it is performed.

Other representatives of GBMs include fullerenes and CNTs. Fullerene is produced by spherically wrapping 2D GR sheets into 0D closed formations [1,30]. CNTs are formed by graphenic layers containing carbon atoms in sp^2^-hybridized state, which are rolled up into a hollow, cylindrical arrangement [31,32,33]. Their important feature is their very high aspect ratio, which is given by their diameter, which is in the range of several nanometers, and length, which can even reach up to millimeters. According to chirality and possible adatoms, they have a metallic or semiconductive character. They can effectively bind biomolecules by hydrophobic interactions, π-π stacking, or van der Waals forces, similarly to GR [1]. Their properties and resulting interactions with biological systems are influenced by many factors, such as the number of layers, the lateral dimensions, shape, purity, and density of defects [34]. A huge number of scientific articles have been devoted to these materials and are therefore not included in this review.

**Figure 5:**
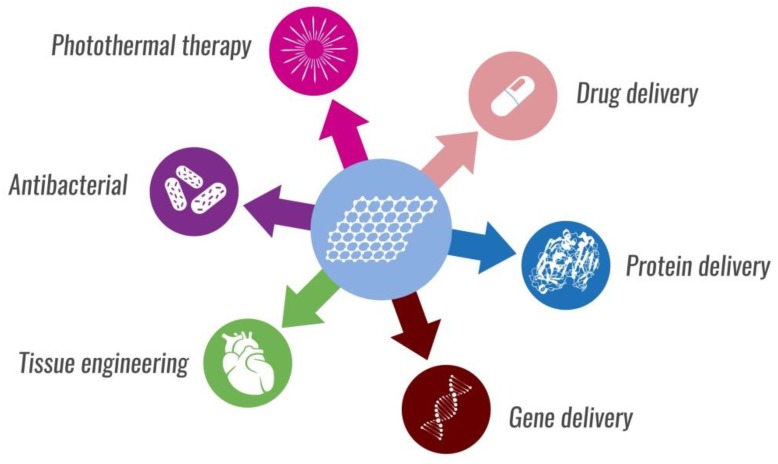
Application of graphene-based materials (GBMs) in the field of biomedicine.

GBMs have also received great attention in the field of biomedicine due to their exceptional features and possible applications (Figure 5) [4].

The proper characterization of materials used to explore possibilities outside of biomedical technologies is one of the most important steps. The main reason for these proper characterizations is that the materials are prepared in scientific laboratories as well as by different manufacturers, using different procedures, which differ in their quality. This is further reflected in the quality of the resulting material. Materials should be described as single-layer GR, multi-layer GR, ultra-thin graphite, GR nanosheets, and GR nanomaterials. According to Wick et al. [35], a few-layer GR is composed of 2–10 GR layers composed of flake-like stacks, while ultrathin graphite consists of more than 10 layers, where the thickness is below 100 nm. This should be applied also for GO [15,35,36].

**Figure 6:**
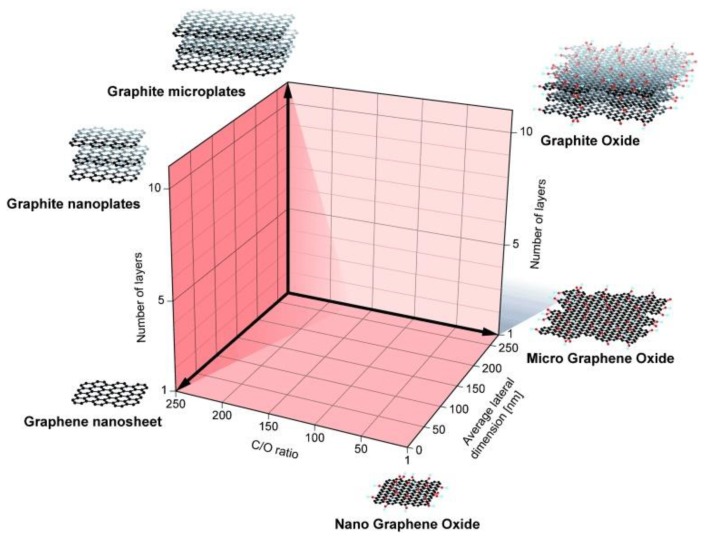
Grid for the classification of GBMs, based on their atomic C/O ratio, lateral dimension, and number of layers. GBMs shown in the corners of the grid correspond to the ideal cases. The grid is in nanoscale, but one can use it for the microscale. Reprinted from [35] with permission from John Wiley and Sons, copyright 2019.

GR- and GO-based materials can be characterized according to the procedure proposed by Wick et al. [35]. The basic qualities which should be carefully observed are the number of layers (thickness), lateral size distribution, and C/O ratio, as is represented in Figure 6 [16,30,35]. The lateral size of GR flakes is one of the major factors influencing GR properties, because differences in size and geometry encourage a change in the ratio between the edge and the bulky structures, resulting in limited space in specific dimensions and a consequent change in mechanical and electrical properties [37]. This characterization is very important for biomedical applications, because it is obvious that 2D GBMs with rough surfaces, a small size, and sharp edges more readily penetrate cells as opposed to smoother GBMs with larger dimensions. Because each carbon atom lies on the surface, these materials, especially in the case of monolayered GR, have a theoretical maximum surface area. For that reason, they have an exceptionally large capacity for the adsorption of molecules and biomolecules. Their capacity corresponds to the specific surface area and bending stiffness, and is associated with the number of layers. Obviously, more 2D GR layer material has a lower adsorption capacity [3].

Analytical methods, such as scanning and transmission electron microscopy (SEM and TEM), atomic force microscopy (AFM), infrared and/or Raman spectroscopy, X-ray photoelectron spectroscopy, and inductively coupled plasma mass spectrometry (ICP/MS), are the most useful tools for characterization [4,38,39,40]. TEM or Raman spectroscopy and AFM allow the determination of the number of layers and lateral dimensions. An accurate and real view of sheet quality, the number of defects, and delamination achieved can be obtained from AFM, which allows the determination of lateral dimensions by analyzing the shape and height of the GR/GO flakes [15,36,41,42,43].

There are many other methods for characterization for different purposes, for which the prepared GR is used. Among them, dynamic light scattering, zeta potential and optical absorbance measurements, such as UV/VIS spectroscopy, can be listed [44]. For example, UV/VIS spectroscopy can be used to observe changes in GO structure after the functionalization of its surface. GO has a strong absorption of UV/VIS radiation at 230 nm. Characteristic absorbance at 300 nm is done by π→π* transitions of C=C bond and the n→π* transitions of C=O bond, respectively. Upon the binding of the functional groups to C=C bonds, a shift from 230 to 266 nm occurs, and the shoulder at 300 nm is reduced [27,40,45].

## 3. Functionalization

The main disadvantage concerning the applications of GBMs is solubility [46]. In general, materials with GR structures have limited solubility in aqueous and polar media and tend to aggregate through van der Waals interactions and π-π interactions [47]. While GR is a strongly hydrophobic material, GO containing oxygen functional groups is highly stable in water. However, it often undergoes aggregation when transferred to cell culture media or buffers [16,48,49].

Chemical modifications and the functionalization of GBMs are important methods for structural changes in GBM surface, which lead to an increase their solubility in biological systems and allow for tailoring of their interfacial and physicochemical characteristics, such as for the preparation of advanced functional GBMs [47,50]. There are two main groups of functionalization methods, namely, covalent and non-covalent functionalization. The first group of functionalization methods is characterized by the surface oxidation of carbon and the binding of organic/inorganic ions/atoms/molecules via covalent bonds, using atoms (fluorine or nitrogen), functional groups (hydroxyl, amino or carboxyl groups), biomolecules, or polymers, at end-caps and defect sites [32,51]. Consequently, the interfacial interactions of GR are highly enhanced. Typical reactions of covalent functionalization are nucleophilic substitution, radical and electrophilic addition, condensation, cycloadditions, and further derivatizations with large number of molecules or functionalization using cationic, anionic, and radical polymerization [1,36]. However, covalent functionalization alters the structure of a GBM. Functional groups form covalent bonds with the GR lattice, and the hybridization of some carbon atoms in the sp^2^ state is changed to sp^3^. This leads to disruption of the translational symmetry of GR and changes the electronic and transport properties of functionalized GR [52].

Covalent functionalization can be used not only for GR, but also in the case of GO and rGO to tailor their properties [53]. However, the present oxygen-containing groups have high chemical reactivity. Therefore, it is difficult to control the functionalization process, since different reactions may occur simultaneously [50]. Generally, the process enhances solubility and the dispersion of GBMs in different solvents, reduces aggregation, and also creates a gap between the conduction and valence bands, because the Fermi level is shifted either above or below the Dirac point [46,54].

Interactions between the delocalized π-electron system on the GR surface and structures on the functionalizing moieties are the basis of noncovalent functionalization. These interactions include electrostatic interactions, π-π stacking, van der Waals forces, hydrogen bonding, and coordination bonds. Here, the GR pristine structure is not destroyed and its native properties are maintained, however the forces between the GR surface and the functionalizing molecules are weak. This technique is not satisfactory for applications requiring strong interactions [55]. The interfacial properties can be adjusted by various polymers or surfactants. Among commonly used surfactants are sodium dodecyl sulfate, sodium dodecyl benzene sulfate, gum arabic, Triton X-100, and cetyltrimethylammonium bromide [46].

Functionalization is used, for example, to modify GBMs, which then carry biochemical signals that instruct cells to heal [56]. GO or rGO are covalently functionalized through their oxygen-containing functional groups to install bioactive molecules. Eckhart et al. used chemically altered GO for binding hydroxyapatite-mimicking polyphosphate groups accompanied with calcium cations that stimulate osteogenesis in vitro and support ectopic bone formation in vivo [56]. Peptide-GR conjugates are examples of functionalization in which the biological activity of the peptide used is combined with the exceptional properties of a GBM [56]. Pluronics™ are triblock copolymers with a back-bone composed of (poly(ethylene oxide)-block-poly(propylene oxide)-block-poly(ethylene oxide). The functionalization with Pluronic™ results in GR with sufficient colloidal stability, because the fragment of poly(propylene oxide), as a hydrophobic part, interacts with the hydrophobic surface of GR, while poly(ethylene oxide), as the hydrophilic fragment, interacts with water [57]. Amine-terminated PEG can be linked to the carboxyl groups of GO through amide bonds. The PEGylated GO is highly dispersible in water, as well as in more complex aqueous solutions, like serum or cell mediums. Polyvinyl alcohol forms ester bonds directly with GO carboxyl groups, or the carboxyl groups can first be transformed into acyl chlorides and then esterified [13].

Functionalization by metallic NPs is another promising possibility for surface treatment. At present, the research of GR decorated with NPs is one of the most intensively studied areas. The main advantage of anchoring metal NPs on GR plates is an increase in electrical, electrochemical, optical, and thermal properties. The synthesis of Ag, Au, Cu, and Pt NPs of homogeneous size, as well as Au/Ag/Cu/Pt alloys embedded on GBMs surfaces, is usually performed by the chemical reduction of cationic metal ions (Ag^+^, Au^3+^, Cu^2+^, and Pt^4+^) [52,58].

## 4. Cytotoxicity and Biocompatibility

One definition of biocompatibility is that biocompatibility is the ability of materials to interact with the living body, tissues, and cells without harmful effects [47,59]. GBMs show both possible effects on biological cells, where in some cases they are biocompatible, and in others they are toxic to cells. While graphite is a well-researched, naturally occurring carbon allotrope, GR, GO and rGO are man-prepared materials with insufficiently known effects on biological systems [1]. Their distribution in organisms, excretion, and toxic effects are determined by their reactions with the biological system. Previous studies have shown that the information obtained on the in vitro and in vivo toxicity of GBMs due to their dimensions and variation in oxidation states is far from complete [60]. The interactions of living cells with GBMs depends on their hydrophilicity, surface chemistry, purity, lateral dimensions, layer number, and dose [61,62,63,64]. These properties vary greatly according to their synthesis eventually to methods of functionalization.

It was proven in several studies that GR NPs increase the number of reactive oxygen species (ROS) in cells, leading to harm to DNA, lipids, and proteins. Likewise, GR increases the rate of the cell injuries, apoptosis, and necrosis, resulting in autolysis joined with the premature death of tissue cells. The cellular toxicity of GR is caused by its agglomeration on cell membranes. The sharp edges of the GR can act as knives and cut or penetrate the cell membrane, causing physical damage [65,66,67,68,69].

GBMs can affect cell membranes and damage them with subsequent cytotoxic effects. Cell membranes contain phospholipids, which are composed of a head group based on phosphate and two chains of fatty acids [61]. The head groups are formed by phosphatic acid modified also by small molecules such as glycerol, inositol, ethanolamine, serine, or choline, which give rise to the phospholipid distinctive properties. There are also cholesterol molecules present, for stabilizing the membrane structure, maintaining fluidity, etc. Pristine GR is not able to electrostatically bind phospholipids, due to lack of charge in its lattice, but it can hydrophobically interact with long chains of the fatty acids. Membranes can be also damaged by withdrawing cholesterol molecules from the membrane [61]. On the opposite side, GO can electrostatically interact with membrane lipids due to the presence of oxygen-containing functional groups on its surface, contributing to a high negative charge density. Finally, GO can directly damage the cell membrane by hydrophobic interaction without entering the cells.

In the comparison with cell membranes at physiological pH values, due to the presence of phosphatidylcholine liposomes of the cells, the total negative charge (ca. −20 mV) is provided [70]. In addition, the cytotoxicity depends on the charge on the surface, as mentioned below in Section 7. Negatively charged nanoparticles show lower unfavorable effect on the cells viability because of the negatively charged cell membrane (ca. −20 mV), which plays an important role to separate the cytoplasm from the outside environment [71]. The positively charged surfaces are more effectively adsorbed on the cell membrane as compared with the negatively charged or neutral ones. However, the positively charged surfaces cause the plasma membrane disruptions, as has been reported [72].

**Figure 7:**
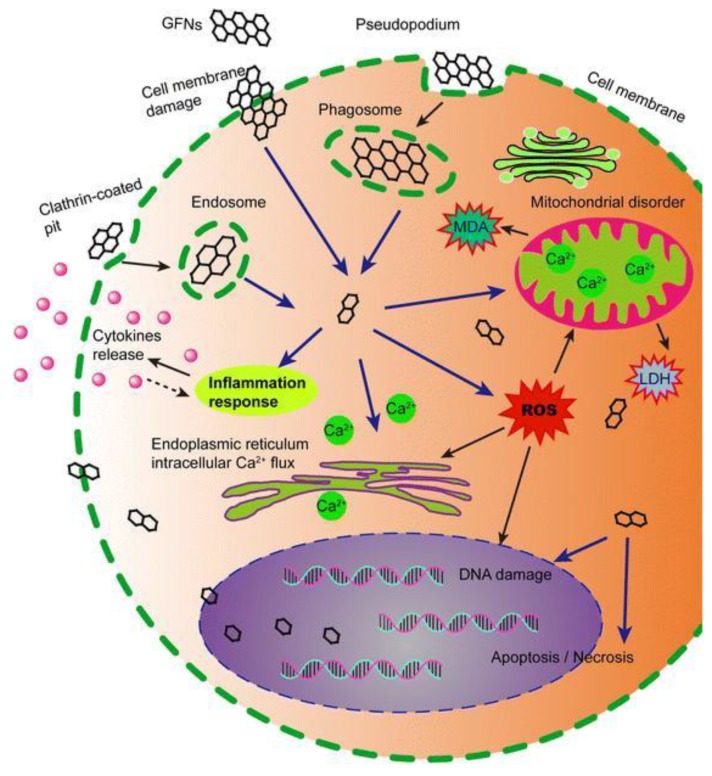
Mechanisms of cytotoxic action of GBMs on living cells. Reproduced from [65], with permission from SpringerLink, copyright 2019.

However, GBMs also enter cytoplasm, as they possess a small size and sharp edges. Their penetration through the cell membrane damages the membrane, and cytoplasmic content is spilled out of the cell. Their toxic effects are manifested by mitochondrial disorders, ROS, and, consequently, lipid peroxidation. If they penetrate the nucleus, GBMs can react with DNA and produce genotoxic effects [61,65]. Figure 7 shows examples of the mechanisms of cytotoxic action of GBMs on living cells [65].

GO shows significant antibacterial activity against gram-positive and gram-negative bacteria, and, at a certain dose, it also presents cytotoxic effects in human cells [73]. GO can induce granuloma formation in the spleen, kidneys, liver, or lungs, and it is not eliminated by the kidneys [67]. Although there have been many demonstrations of possible applications of GO for biomedical purposes, the lack of live cell compatibility information has curbed the scope of GO applications [73]. Cytotoxicity can be reduced using, for example, chitosan (CS) attachment onto GO surfaces [73].

A few studies have been conducted to compare the biocompatibility of GO and rGO, and the results are contradictory [74]. Most studies claim that rGO-based materials are less toxic than their parental GOs. Apparently, due to reduction of functional groups, they can have interactions with biomolecules due to their higher hydrophobicity. Other studies have shown that rGO is more harmful to some cells, such as U87 and U118 glioma cells, compared to GO when inducing their apoptosis [74].

In one study [67], the cytotoxic effects of GR on A549 human lung epithelial cells was investigated. The MTT assay was used for tests of viability of the cells and the results were statistically assessed to find the dependence between the concentration and time variables and the GR-induced death of cells. The GR deleterious effects in A549 human lung epithelial cells have been shown to be time and dose dependent. The highest cytotoxicity was observed after exposure for 72 h. The concentrations corresponding to no observed adverse effect and half-maximal (50%) inhibitory concentration were determined to be 40.653 and 0.059 µg/mL, respectively [67].

rGO was stabilized using Pluronic™ P-123 (rGO-P) [57]. However, the dose-dependent cytotoxicity of rGO-P was found in differentiated PC-12-like neuronal cells after exposure for 24 h, manifested by membrane disruption and cytoskeletal integrity. Reactive oxidative stress in PC-12 was confirmed as ROS production increased with increasing dose and prolonged exposure time. Acute toxicity was evaluated in vivo using mice following the administration of rGO-P at 10 mg/kg body weight, and any visible abnormalities in behavior and motor function, as well as other morphological changes, were not observed [57].

The in vitro toxicity of GR and GO in three different sizes of (indicated as small, medium, and large) on HEK 293T cells was assessed. It was observed that the large and small sizes of GR, as well as GO, evidently diminished the viability of the cells and enhanced DNA damage. The process occurred along with the activated generation of ROS, and various associated genetic markers expressions were induced [60]. In addition, high GR and GO acute toxicity on Tox2 bacteria was observed. There was a higher harmful effect of GR than GO. Moreover, this depended on the particle size. Larger particles had stronger effects than smaller particles. In this study, it was summarized that GR significantly reduced survival rates and exhibited acute toxicity, and GO apparently induced DNA damage, ROS generation, and abnormal gene expression. The induced toxicity was influenced by physical properties, mainly the oxidation state and size, as well as concentrations of exposure, in both in vitro and in vivo assays [60].

The main barrier protecting the human body from damaging effects in the surrounding area is human skin. It will probably react first when it comes into direct contact with GBMs. Therefore Frontinan-Rubio et al. studied the effects of GO and GR on human HaCaT keratinocytes at the molecular level on cell, metabolomic, Ca^2+^, and ROS processes, as well as cell motility and death [62]. They observed different effects of GBMs on HaCaT cells and varying amounts of metabolites occurring in biochemical processes, for example, in butanoate metabolism, glycolysis, and the citric acid cycle. This was probably related to variations in the global state of cell bioenergetics affecting mitochondrial function.

The cytotoxicity of pristine GO and GO, modified with ammonia (GO-NH_2_), in cancerous cells A549 of human lung tissue and human non-tumor embryonic stem Lep3 cells after exposition to five particle concentration levels (0.1, 1, 10, 20, and 50 µg/mL) for 24 and 48 h was evaluated [63]. Characterization of the prepared GO-NH_2_ particles showed they possess a higher thickness but smaller size, with a positive charge of surface and an increased ability to aggregate in cell cultures compared to GO. It was concluded that both tested materials exhibit different cytotoxicities, which is different for cells and depends on the dose and time of exposure. Obviously, the particles of GO-NH_2_ were more cytotoxic than pristine GO. The non-tumor embryonic stem cell morphology was strongly influenced, whereas cancerous A549 cells better withstood short-term exposure. After 48 h of exposure, the embryonic stem cells proliferative ability remained unaffected and, on the contrary, a strong effect on A549 cell proliferation was demonstrated [63].

Another extensive study has been performed to observe the in vitro and in vivo preclinical biocompatibility of GBMs [47,64]. While in vitro biocompatibility assays generally consist of tests of prokaryotic and eukaryotic cellular cytotoxicity, hemocompatibility, and inflammatory responses, in vivo tests focus on the pharmacokinetic processes associated with GBM absorption, distribution, metabolism, and excretion applied to organisms such as zebrafish, rats, mice, rabbits, or canines. It can be concluded that the physicochemical properties of GBMs (functional groups present on the planar surface, charges, structural defects, and size) contribute to determination of cellular biocompatibility. It seems that structures with a smaller size and a higher degree of oxidation improve material biocompatibility. GBM functionalization with macromolecules, such as PEG, proteins, dextran, and CS, enhances the biocompatibility and attenuates cytotoxicity [47,64]. The opposite results found in a cytotoxicity and biocompatibility study may be due to different assessments of the methodologies used. It can be concluded that well-defined, long-term toxicity studies are needed to increase confidence in these materials for biomedical applications [47].

## 5. Nanotheranostics and Tissue Engineering

The combination of therapy and diagnosis is called nanotheranostics. It involves combining diagnosis and therapy in a single platform based on the use of nanomaterials [75]. It allows early detection of diseases, resulting in more effective treatment. Antitumor drug targeted delivery is made possible by the use of non-toxic nanosized carriers, using both active and passive transport mechanisms. GBMs belong to the standard nanocarriers, together with micelles, dendrimers, liposomes, stimuli responsive cargos, nanocantilevers, quantum dots, magnetic NPs, metal NPs, polymeric NPs, nucleic acid based nanomaterials, etc. GBMs are very promising in this context, as the application of metallic NPs is limited due to their stability in cells, surface chemistry, and cytotoxicity, as well as due to their accumulation at lymph nodes, adrenals, spleen, liver, kidneys, and bone marrow.

Tissue engineering is among the prominent therapeutic approaches connecting multiple scientists of different scientific branches. Nowadays, designing an appropriate scaffold for supporting cell growth, with a sufficient mechanical stability to complete a process of regeneration and maintain surgical placement is an interesting task, especially in the case of the repair of cardiovascular and orthopedic injuries [76].

Research into the use of GBMs in tissue engineering, as well as regenerative medicine applications, is only beginning, however, it seems to be moving forward quickly. GBMs, including CNT and GR nanosheets, are probably the most popular among all nanomaterials that have been designed for the controlled growth of stem cells. They have been already studied in cartilage, cardiac, neural, musculoskeletal, bone, and skin/adipose tissue engineering [9,47].

**Figure 8:**
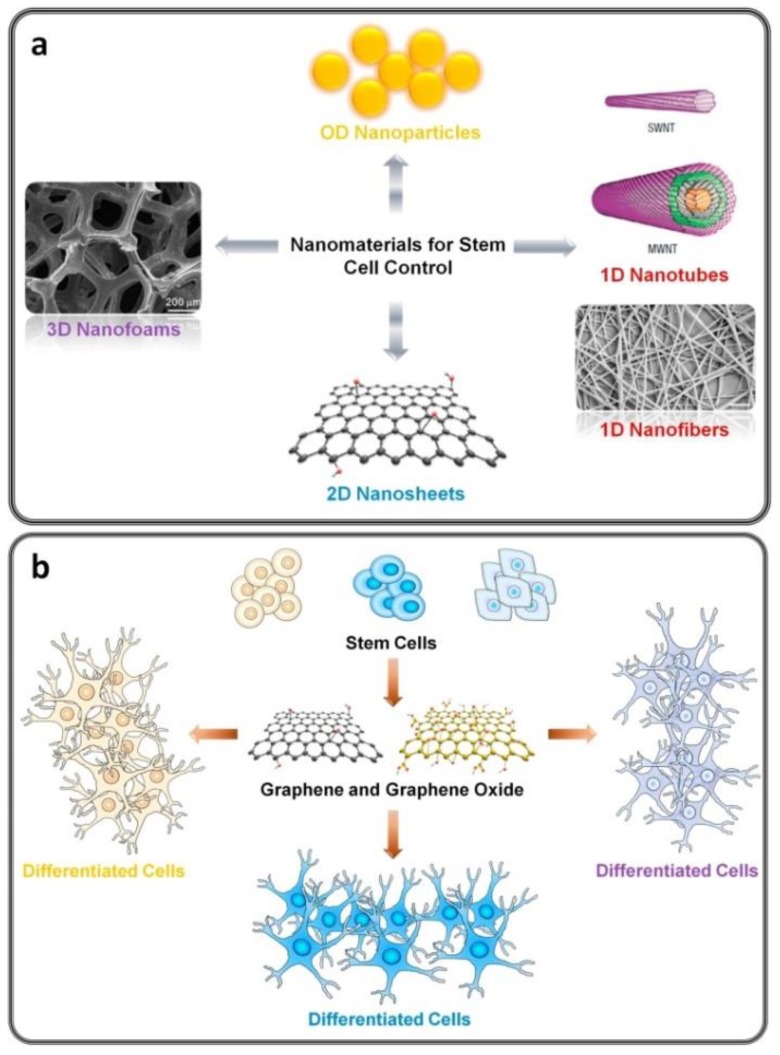
Nanomaterials for the selective control of stem cell growth and differentiation (**a**). Scaffold materials based on graphene (GR) and GO for the culture of various stem cells, multipotent mesenchymal stem cells (in the upper part in left, colored in yellow), neural stem cells (in the upper part, in the middle, colored in blue) and induced pluripotent stem cells (in the upper part, colored in light blue) (**b**). Reproduced from [77], with permission from Elsevier, copyright 2019.

Their superior electrical conductivity and amazing mechanical properties makes them excellent materials for the design and manufacturing of scaffolds for artificial tissue engineering [77]. They can be applied in cell growth and differentiation, in stem cell engineering, and wound healing, or in regenerative medicine. They also appear to be suitable for applications as a reinforcement material for the preparation of fibers, films, foams, hydrogels, and other tissue engineering scaffolds. Figure 8 represents nanomaterials which are used for various stem cell control growth and differentiation methods. Among others, GR and GO have been found to enhance the growth, proliferation, and differentiation of stem cells, such as somatic adults stem (induced pluripotent stem cells) or neural and mesenchymal stem cells (multipotent stem cells), into specific tissue lineages [77].

GBMs have a number of outstanding properties which make them very suitable materials for regenerative medicine, especially for growth of stem cells, such as their proliferation and differentiation ability. They are also for suitable for tissue engineering [56,76]. These properties include, for example, very good chemical stability, a large specific surface area, exceptionally good mechanical properties, such as perfect elasticity or rigidity in the plane, flexibility, high adaptability to flat and irregular surfaces, exceptional electrical conductivity, the ability of mimicking biological tissue properties, and possibilities of functionalization. They intrinsically support cell adhesion, however, biocompatibility is affected by the particle size and surface functionalization type. By using them, tissue regeneration can be performed in a controlled way. They can be functionalized with biocompatible substances such as poly(acrylic acid) (PAA), dextran, or PEG. Modifications with these agents are made for the safer implementation of GBMs in biomedical applications, where they are inert and not related to the treatment process [56].

GR and GO have been studied as substances for bone and neural tissue engineering. It was found that the differentiation of the stem cells was facilitated in GO because oxygen-containing functional groups on its surface allow intermolecular interactions (electrostatic attraction, π-π interactions, or hydrogen bonding) [78].

Pristine single-layer GR cytotoxicity, as well as the possibility of its application for the scaffold preparation for the L929 mouse fibroblast cell line, was studied in [79] by observing cell viability, cell adhesion, morphology, cytoskeleton architecture, and movement to the scratch-wound area. It was confirmed that GR was not cytotoxic for the L929 cell line and conversely enhanced the adhesion of cells and their proliferation within 24 h [79].

Microribbons based on the NC poly(lactide-co-glycolide)-graphene (PLGA-GR) were prepared for neural tissue engineering, as was described in Aval et al. [80]. Different GR concentrations in the NC and the associated effects on the mechanical, physical, chemical, and biological characteristics were studied. GR layers were aligned in the PLGA matrix as roughness grooves on the microribbon surface. Hydrophilicity, electrical conductivity, tensile strength, and the elastic modulus of the PLGA-GR microribbons were all enhanced due to the incorporation of the GR nanosheets. Compared to PLGA, a higher rate of differentiation of SH-SY5Y cells into mature neurons was achieved with PLGA-GR. Thus, neuroblastoma cells were grown using this PLGA-GR scaffold, showing great potential for central nerve regeneration [80].

In other work, the effects of GO derivatives on the biological, mechanical, physical, and chemical characteristics of CS-gelatin (CS-Gel) scaffolds were studied. The polymeric chains of CS and gelatin were modified with GO and amine-modified GO (G-NH_2_) via the covalent bonding of amine and carboxylic groups. The physical and chemical properties of scaffolds, such as their density, porosity, shape retention, interconnectivity, and capability of water absorption and retention were influenced by surfaces with positive and negative charges of both GO and G-NH_2_ nanosheets compared to the original non-functionalized CS-Gel scaffold. The mechanical properties of functionalized scaffolds and cell viability were improved by 4–10% [76].

The biological, mechanical, physical, and chemical characteristics of scaffolds based on CS-Gel were observed after adding GO and montmorillonite (MMT) [81]. Their presence enhanced water absorption and retention, although the porosity of modified scaffold was lower. Compared to the CS-Gel scaffold, a significant improvement in CS-Gel/GO/MMT mechanical characteristics was observed. The compressive strength and Young’s modulus were increased from 0.14 to 0.23 MPa and from 17.56 to 22.73 MPa, respectively. Compared to the CS-Gel scaffold, the adsorption of protein increased twice in CS-Gel/GO/MMT. The modified samples did not show any cytotoxicity and cell viability was improved by 30% after 72 h of incubation [81].

One of the biggest health problems in the world is bone damage caused by traumas and sports related injuries, as well as congenital defects [82]. A NC scaffold of gelatin, alginate, and GO (in varying concentrations) for improving bone regeneration was prepared and tested. Compared to the scaffold based on alginate and gelatin without GO, the compressive strength of the NC scaffold after the incorporation of GO increased significantly. The NC was highly hydrophobic (ca. 700% of swelling) and slowly biodegradable (ca. 30% in 28 days). Confirmation that the proposed scaffold may be a suitable osteoinductive material was performed using in vitro studies on MG-63 cells and tests of cell differentiation using the mesenchymal stem cells. These tests showed an increase in both cell adhesion and proliferation [83].

Magnetic graphene oxide (MGO) decorated by NPs of Fe_3_O_4_ was prepared to study cell behavior and the course of osteogenic differentiation, along with the mechanism of the MGO-induced effect, using mesenchymal stem cells derived from rat bone marrow (BMSCs). In conclusion, it was found that MGO, at low concentrations, is biologically compatible and simultaneously notably accelerates osteogenic differentiation in BMSCs [84].

Many new tissue engineering strategies have been proposed that largely imitate the biological properties of body tissues that are very complex and reproducible. Hybrid scaffolds of decellularized tissue (DT) and GO were presented in [85]. The GO concentration effects on the physical, chemical, and structural characteristics, such as the porosity, pore size, morphology, mechanical strength, and capacity of water uptake, have been tested. The chemical treatment of a bovine heart was used for DT preparation. Compared to the DT scaffolds, the scaffold containing 3% GO showed a mechanical strength and cell viability increase of about 25%. This porous scaffold promoted cellular activity and cell adhesion and proliferation using a biomimetic construction suitable for clinical applications [85].

Hydroxyapatite, which is used as a material for bone tissue replacement, is commonly applied for bone defects treatment due to its brilliant biocompatibility. Nevertheless, there are limitations for its applications due to difficulty of processing and its poor osteoinductive ability. Zhou et al. provided a method of a soft template for developing a porous scaffold characterized by a hierarchical pore structure, which is useful for cell adhesion and ingrowth, fluid transfer, demanded porosity, pore size, and morphology. The material also possesses sufficient biomechanical strength. The addition of rGO promotes adhesion and proliferation, as well as the spontaneous osteogenic differentiation of mesenchymal stem cells derived from bone marrow. Therefore, this porous hydroxyapatite/rGO composite scaffold is capable of promoting bone growth in scaffolding and repairing severe bone defects [86].

## 6. Biosensors

Biosensors based on GBMs are adaptable diagnostic tools that possess faster detection than traditional methods. They can detect femto- or pico-molar concentrations of measured substances [87]. A chemical sensor is an instrument which is able to quantitatively/semi-quantitatively convert the detection of a chemical into an evaluable signal [9]. Generally, these sensors are formed by a receptor and a transducer. Any organic or inorganic substance capable of interacting with one species or group of species can be used as the receptor. The transducer transforms chemical information into a signal. Biosensors can be classified according to the transducer system as field-effect transistors, resonant biosensors, optical-detection biosensors, ion-sensitive biosensors, electrochemical biosensors, and thermal-detection biosensors. Among others, optical sensors, on the basis of metal NPs or chromophores, or electrochemical and surface plasmon resonance (SPR) sensors, based on metal NPs, should be named as well [9].

Based on our knowledge, GBMs are suitable candidates for the sensing of biomolecules due to their electrical, structural, and thermal properties.

Many pathological processes in various tissues and organs are manifested by a change of a condition in the body, such as a change in pH, temperature, ion concentration, redox state, biomolecules, and more. The development of probes for the tracing of these changes can contribute to the explanation of intricate biological processes [9,88]. The sensing of glucose, DNA, RNA, dopamine, the cytochrome complex, intracellular redox state, and pH sensing in cell microenvironments can be among biochemical and biophysical parameters which need to be monitored [78,88,89,90,91,92,93]. They can be used for the early diagnosis of lung cancer, breast cancers, and ovarian carcinomas, or for the determination of cystadenocarcinoma carcinoembryonic antigen (CEA). The monitoring of CEA using electrochemical immunosensors based on GR derivatives (frequently rGO) has been described, for example, using rGO functionalized by AuNPs and poly(l-arginine) [78].

**Figure 9:**
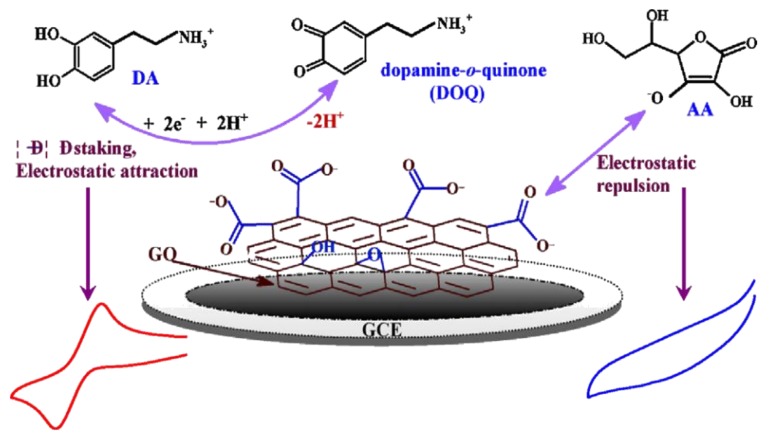
Estimated electrochemistry of dopamine (DA) and ascorbic acid (AA) on a glassy carbon electrode (GCE), modified using graphene oxide (GO/GCE). The red and blue curves (the bottom part in left and right, respectively) correspond to the electrochemical signals of DA and AA, respectively. Reproduced from [93], with permission from Elsevier, copyright 2019.

For example, Gao et al. studied the electrochemical interactions of dopamine (DA) and ascorbic acid (AA) on a glassy carbon electrode (GCE) modified using GO [93]. They described a difference in the electrochemical behavior of DA and AA on GO/GCE. It can be estimated that DA molecules, which have a positive charge and aromatic character, easily interact with the GO/GCE interface via π-π bonding and electrostatic attractive forces due to the GO surface, resulting in the production of a large electrochemical response. AA is characterized by its non-aromatic character and does not allow π-π stacking with GO. In addition, there is electrostatic repulsion between the GO surface and AA, preventing interactions between AA and the transducer interface. This leads to the electrochemical signal completely disappearing. Figure 9 shows a suggestion of the electrochemical behavior of dopamine (DA) and ascorbic acid (AA) on a glassy carbon electrode (GCE) which was modified using GO [93].

An advantage of GO is the presence of its hydrophobic and hydrophilic properties, which have allowed the development of novel sensing methods, such as electrochemical detection, surface-enhanced Raman spectroscopy, fluorescence resonance energy transfer-based biosensors, and laser desorption coupled with ionization mass spectrometry [89,91].

GR-based biosensors include enzymatic electrochemical biosensors that have enzymes immobilized on the electrode surface and allow biomolecule detection. They are also applied in conducting electrochemical immunosensor studies where antigen-antibody complexes are detected on the electrode surface.

Thus, biosensors are of paramount importance biosensors due to their properties. Research on a highly sensitive biosensor can provide new materials, which is useful for the very sensitive determination of various biomarkers and compounds in various areas of life science, such as biomedical, food, agricultural, and environmental applications, along with equally important applications in industry [87].

## 7. Antibacterial Properties

Similarly to various NPs, such as CNTs, metals, metal oxides, metalloids, and CS NPs, GR-based NCs have intrinsic antimicrobial properties [92,93,94,95,96]. It has been confirmed that surface-immobilized GR nanoplatelets on silicone rubber peritoneal dialysis catheters possess a strong antimicrobial effect against *Staphylococcus epidermidis* [97]. Thin films and porous membranes containing polylactic acid (PLA), carvacrol (CRV, oregano essential oil, having antibacterial, antifungal, antioxidant, and anticancer activities), and GR nanoplatelets were made by electrospinning and solvent casting using various formulations. CRV releases, as a function of time, as well as the release mechanism, were studied. The results demonstrated that the incorporation of GR nanoplatelets was associated, while reinforcing, solidifying, strengthening, and maintaining sufficient ductility. Furthermore, the integration of GR nanoplatelets allowed the adjustment of the amount and kinetics of the CRV liberate and decreased the activity of the initial burst liberate [98].

Opposite observations, from the inhibition to the increased growth of bacteria, were described as mechanisms of interaction between GO and pathogens. The influences of GO against human pathogens were summarized. In approximately 27.8% of cases, no antibacterial effect was observed, while in approximately 66.6% of cases a bacteriostatic effect was shown. The morphology, GO size, concentration, exposure time, incubation protocol, and microorganism type are properties that affect the interaction between GO and bacteria [99,100,101]. Many studies have verified the antibacterial efficacy of GO in the form of freestanding paper, nanosheets, or nanowalls. It has been supposed that the antibacterial activities are mediated by the physicochemical interactions between GO and microbial agents. Three main mechanisms of actions were suggested, namely, nanoknives due to sharp edges, oxidative stress, and the wrapping or trapping of bacterial membranes by flexible, thin GO films. GO, a new antibacterial material, is preferable to other carbon-based nanomaterials, especially CNTs, due to its low toxic effects and compatibility. Well-dispersed, freestanding GO nanosheets have been demonstrated to have the highest antibacterial effect among several GR-based nanomaterials. Here, the antibacterial activity of GO coatings against *Escherichia coli* showed higher activity than that against *Staphylococcus aureus*. The sharp edge of GO, which was at a perpendicular position to the bacteria, caused greater damage to *S. aureus* in direct contact than to *E. coli* due to the absence of any additional outer membrane in the case of *S. aureus*. However, *E. coli* was more damaged by the GO coatings than *S. aureus*. Taking into account the structure of bacterial cell wall, oxidative stress from GO more easily penetrated through the thinner cell wall of *E. coli*. The disruption of the cell by GO coatings without sharp edges was further confirmed by a change of bacterial morphology after exposure to the coating. The membrane integrity of the cell was lost, and cytoplasm was leaked, resulting in final cell death. Considering the morphological properties of GO coverings, in particular, that they are edge-free and bonded in parallel to the given substrate, the ROS mechanism was considered as the principal factor of antibacterial activity [100].

**Figure 10:**
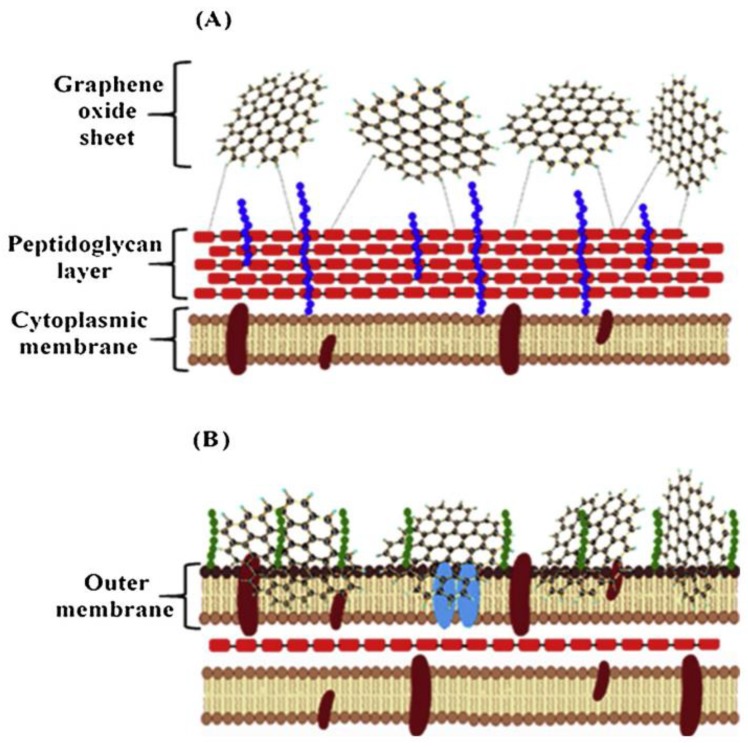
Design of the possible mechanism of GO interactions with gram-positive and gram-negative bacteria. (**A**) Mechanical wrapping in gram-positive bacteria and (**B**) the damage of the membrane in gram-negative bacteria. Reprinted from [102] with permission from Elsevier, copyright 2019.

The antibacterial properties of GO have evoked broad concern in various medical branches, in spite of the exact antibacterial mode of action of GO not being discovered thus far. While investigating the interactions of GO with gram-positive and gram-negative bacteria, it was ascertained that the transformation of GR to GO and the associated antimicrobial effects of GO are concentration and time-dependent. The loss of bacterial membrane integrity increases with rising GO concentrations, which conform to the increased release of lactose dehydrogenase in the medium. Differences in the GO mode of action in relation to gram-positive and gram-negative bacteria were apparent with cell captures, which were mainly with gram-positive *S. aureus* and *Enterococcus faecalis*, while membrane contravention, because of physical contact, was found for gram-negative *E. coli* and *Pseudomonas aeruginosa*. According to ATR-FTIR characterization, changes in the fatty acids, amides I and II of proteins, peptides, and amino acid regions of GO-treated bacterial cells were observed when compared to untreated bacterial cells. Thus, these data further explain the antibacterial effect of GO against bacteria. Figure 10 represents possible interactions of GO with membranes of gram-positive and gram-negative bacteria strains [102].

Hydrogels [103,104,105], especially those with a combination of GO and PEG, are characterized by good biocompatibility and an intrinsic antimicrobial activity, and are perspective colloidal building blocks for the fabrication of 3D materials, which are increasingly used in the encapsulation and delivery of drugs and cells. Cheng et al. prepared a colloidal injection hydrogel composed of GO. The reversible folding of this product is due to pH-dependent hydrogen bonds. The formulation has high antibacterial activity and possesses a good drug release ability [106].

Rasoulzadehzali and Namazi demonstrated the antimicrobial efficacy of pH-sensitive bio-NC hydrogel beads made of CS and GO-Ag nanohybrid particles for the controlled release of doxorubicin (DOX) towards gram-positive and gram-negative bacteria, studying the anticancer effect as well [107]. A GO-CS-Ag NC, fabricated by the agitation of CS with GO nanosheets synthesized using Hummers’ method, with Ag NPs fabricated from a green tea leaf extract, was tested against *S. aureus*, *Streptococcus mutans*, *E. coli*, *Klebsiella pneumoniae*, *P. aeruginosa*, and *Salmonella typhi*. In addition, the antibacterial activity of the starting materials and intermediates was investigated. The nanomaterials demonstrated significant antibacterial potency, efficiently inhibiting the growth of pathogenic bacterial strains. However, the antibacterial effect decreased in the following order: GO-CS-Ag > GO-Ag > GO-CS > GO. This result suggests that the newly developed GO-CS-Ag nanosystem can potentially be applied in biomaterials for biomedical and food purposes [58].

Antimicrobial electrospun mats, fabricated from the dip coating of a PLA nanofiber into a GO covered by dopamine (DMA) (PLA-GO-DMA), have been proven to have an antibacterial effect towards *E. coli* and *S. aureus*. Moreover, the biocompatibility of the mats was studied using HUVECC and the HepG2 and A549 cell lines in PLA-GO-DMA, demonstrating significant biocompatibility [108].

Prepared poly(ethylene glycol-amine)-derivatized GO nanosheets labelled with fluorescein isothiocyanate (FITC-PEG-GO) were effectively absorbed by peritoneal macrophages, causing a considerable increase in the phagocytosis of the important opportunistic fungal pathogen *Candida albicans* by pro-inflammatory M1 and reparative M2 macrophages. Treatment with GO increases M1 macrophage activation, which is considerable for the eradication of pathogens, and decreases alternative M2 macrophage activation, which decreases fungal persistence and inhibits chronic infectious diseases. This higher fungicide potency of macrophages after GO treatment seems to be promising for future treatments [109].

Sandhya et al. prepared rGO via reduction based on potato starch and zinc oxide coated rGO (ZnO-rGO). In this case of ZnO-rGO, the reduction and the conversion of ZnO to nano-ZnO occurs simultaneously. The antibacterial effect of the materials towards *E. coli* was tested. The results demonstrated that ZnO-rGO has better antibacterial properties than rGO, which was attributed to the synergistic effect of ZnO and rGO towards the bacteria in the NC. It was also found that the antibacterial activity of ZnO-rGO against *E. coli* is associated with the disruption of the bacterial cell, which was confirmed by AFM images [110].

Frequently, antibacterially-active nanosystems are used in combination with antibacterially-active drugs [92,95,111]. Multiple mechanisms of action provide the synergistic activity of such combinations, preventing the occurrence of resistant or multidrug resistant strains, or even successfully destroying such pathogens. For example, tyramine-conjugated GO (GOTA)-immobilized TiO_2_ was developed for orthopedic applications, with the purpose of efficiently transferring and releasing antimicrobial agents, preventing implant-associated infection. Subsequently, doxycycline (DXC) was loaded onto GOTA/TiO_2_ via non-covalent bonds. The DXC/GOTA/TiO_2_ NC contained 36 μg of DXC per cm^2^, and it was ascertained in in vitro experiments that the sustained release of DXC from the TiO_2_ surfaces continued for more than 30 days. DXC/GOTA/TiO_2_ demonstrated higher antibacterial activity against *E. coli* and *S. aureus* than bare TiO_2_ and GOTA/TiO_2_, without any damage to the viability of human dermal fibroblasts [112]. Ma et al. investigated the release of ciprofloxacin (CPX) from GO and rGO in the presence of MMT in simulated gastrointestinal fluids. The order of adsorption affinity of CPX was as follows: rGO + MMT > GO + MMT > rGO + MMT + pepsin > rGO > GO + MMT + pepsin > MMT > MMT + pepsin > GO > rGO + pepsin > GO + pepsin. MMT increased the adsorption of CPX on GO and rGO, owing to the hydrated coating of Si on the GO and rGO in the simulated gastric fluid. CPX was adsorbed by GO and rGO and the adsorbed CPX was liberated from MMT into solution by electrostatic repulsion. The reduced CPX ratio was lowest in the GO/rGO + MMT + pepsin system. A mixture of MMT and GO/rGO reduced the CPX concentration in the gastric fluid, which consequently results in the low antibiotic activity of CPX [113].

GO covered with mannose and PEG (GO-PEG-MAN) has been shown, in vitro, to have an enhanced intake of macrophages by using mannose receptor-mediated endocytosis. Rifampicin (RIF) uptake was considerably increased when loaded with GO-PEG-MAN, causing an increase of RIF concentration in macrophages, lasting for a long period, leading to a more elimination of intracellular *Mycobacterium tuberculosis*. Moreover, based on the above-mentioned fact, MAN-GO DDS was suggested for the removal of macrophage ablation in atherosclerotic plaque [114].

Hybrid systems, prepared from the antitubercular agents isoniazid and pyrazine-2-carbohydrazide, covalently linked to GO, were developed to obtain a multi-target hybrid system with enhanced antimycobacterial effects and a broader range of activities, including being effective against bacterial (from the ESKAPE list) and fungal strains. The NCs were tested against mycobacterial, bacterial, and fungal strains, and their microbicidal effect was determined to be caused by the induction of membrane depolarization. The toxicity of the agents to Hep-2 was insignificant at tuberculostatic concentrations. The NCs were able to suppress resistance and enhance the effect of first-line drugs against tuberculosis. The high antimycobacterial efficacy of GO, its synergism with isoniazid, and the main anti-biofilm effect were observed for combinations between GO and the two first-line tuberculostatics [115].

Unfortunately, GO has also been reported to be toxic to mammalian cells. For example, Singh et al. described the thrombotoxicity of GO in mice, also elucidating the evocation aggregation response in human platelets [71]. The surface of GO determined the biocompatibility, which was used in preparation to formulate variously functionalized GO. Amine-functionalized GO sheets demonstrated insignificant cellular toxicity. These opposite properties seem to be connected with the surface charge [116,117,118,119], characterized by, for example, the zeta-potential. Based on the performed measurements, the GO was shown to be negatively charged in the pH range from 3.5 to 9. This was determined to be caused by carboxylic moieties. Amino-modified sheets demonstrated a positive charge when the pH was less than 10. The effect of surface functionalization of GO on bacterial efficacy and toxicity on human cells has also been described, for example, by Valentini et al. [120].

The applications of GR, GO, and rGO as antimicrobial agents are limited (besides frequent mammalian cell toxicity) by their aggregation, which is caused by van der Waals interactions between sheets. Additionally, this effect can be prevented by the functionalization of the surface of nanomaterials. Detailed information on the possibilities and types of modifications of surfaces of GR, GO, and rGO by metals and/or their oxides, metalloids, natural and synthetic polymers, antibiotics, enzymes, and their behavior as solids and in solution have been described by Kumar et al. in their comprehensive review [121].

## 8. Drug Delivery Systems

Delivery systems are specific formulations that are used to transfer/deliver biologically active ingredients/agents in plants or animal/human organisms to a target/specific site/tissue of action, in order to provide a specific release of the active agent upon delivery, or to ensure the stability of the active agent up until it is delivered to the correct location. In biomedical applications, delivery systems are mainly used for drugs, proteins, and genes [104,105,122,123,124,125,126].

GR can interact with proteins in biological fluids, which may result in significant changes of the molecular physical characteristics and cause harmful reactions in the host’s immune system. The chemical properties of GR and its membrane interactions result in direct toxicity not only to the target but also to neighboring cells. Given the benzene structure of the hexagonal components of the lattice, it can be considered a huge aromatic poly molecule or intact GR, where π-π and hydrophobic forces are the main source of drug binding, however, for other forms of GR, the presence of functional groups provides more possible interactions. Because of this, the use of non-functionalized GR in biomedical applications is limited and other forms based on GR are used [127]. The unstable structure, negative charge, and poor cellular uptake of genes make the usage of a carrier necessary for their protection and transport. GR NPs covered by guanidine and amine moieties possess better potential for coupling with genes (i.e., in DNA or RNA) [27].

Targeted drug delivery can improve the bioavailability, biocompatibility, and safety of therapeutic agents [104,105,123,124,126,128]. This can help solve the problems connected with the treatment of cancer, by reducing the systemic “intrinsic” toxicity of the drug itself (which is a limitation factor of therapy, due to the size of the dose) and, in such a way, overcome undesired side effects [104,105,123,127]. DDSs can also help to overcome the resistance of the cells to the drug [92,104,105,123]. Ideal DDSs are able to deliver a drug to the site of action, control the sustained release of the drug, and protect the drug from degradation. Such DDSs have suitable physicochemical properties that allow their transport in the body, while their specific properties facilitate cell-specific absorption and delivery [104]. Recent trends favor highly-customized NPs, with the control of each physicochemical property, such as the particle size and shape, zeta potential, specific surface area, etc. Nano-carriers increase drug concentrations in cancer cells using both passive and active targeting strategies. This reduces the toxicity to healthy cells. Passive targeting is enabled by increased permeation and retention. Active targeting utilizes specific interactions between the drug carrier and target cells. Nano-carriers are often coated with a variety of ligands that bind to cancer-specific receptors, thus allowing systems to enter the cell through receptor-mediated endocytosis. Covering the NP surface with a biocompatible polymer (e.g., PEG) extends the blood circulation time by preventing opsonization and decreasing absorption by the reticuloendothelial system. Thus, increasing the distribution of the NP into the target tissue is realized, which gives rise to the capability of functional groups (e.g., bis-amine) on the surface to be conjugated with other ligands [129]. Some ligand-receptor targeted DDSs have been studied, such as interleukin, lipoprotein, Fc receptors, lectin, complement, transferrin receptors, and receptors (e.g., dopamine) expressed in, for example, human colon adenocarcinoma and breast cancer. Thus, it is possible to efficiently deliver anticancer drugs through nanosupports and overcome the overall toxicity problems using active and passive targeting mechanisms [34,35]. In addition, various nanosystems can ensure higher efficacy and a lower recurrence rate for combined therapy, including the use of radiation therapy, photodynamic therapy (PDT), photothermal therapy (PTT), and chemotherapy. For combined therapy, multifunctional nanomaterials are becoming of interest due to their effective ability of drug loading, targeted delivery, and controlled drug release [123].

DDSs include nanostructures or nanomaterials such as liposomes, micelles, branched polymers, dendrimers, microspheres, polymer shells, magnetic NPs, metal NPs, quantum dots, various other inorganic NPs, and CNTs, GR-based materials, fullerenes, nanodiamonds, etc. [103,105,123].

At this point, multimodal drug delivery needs to be introduced. These are specifically designed NP systems that are capable of providing multiple functions and are not simply “classic” DDSs. Multimodal DDSs are capable of, for example, immune modulation and intracellular drug delivery or, in addition to drug delivery, also serve as various probes for diagnosis or imaging. The condition is that these systems should not be toxic to macrophages [130,131,132]. The multimodal DDSs were born from the idea that biological phenomena including inflammation, angiogenesis, and tissue remodeling are quite complex. The interlinked processes are also regulated by the spatiotemporal manner of biological processes [130]. The factors in the local microenvironment are elaborated and followed by a defined path. For this reason, the single-factor delivery oversimplifies biology and has not been proven to be clinically effective in pathological states. In this regard, the strategies of delivery of several factors to modulate several stages have paved the way for the pathology. The multiple delivery system, including a number of strategies, offers opportunities for a temporal multi-modal release. These strategies are of importance and have been demonstrated as methodologies [133].

GR therapeutics are used for both diagnostic and therapeutic applications [134,135,136]. One of the first biomedical GR utilizations was for drug delivery. GR-based systems are used in drug-based chemotherapy, phototherapy, gene therapy, and radiotherapy. Great effort has been devoted to design NCs that combine the ability to detect cancer cells at an early stage with effective imaging, drug storage, and controllable drug release [13,137]. Photothermal and redox-responsive DDSs have been designed, covering mesoporous silica nanoparticles (MSNs) with GR quantum dots (GRQDs). The MSNs loaded by rhodamine B (RhB) and GRQDs, capped on MSNs as gatekeepers, can inhibit the loosening of RhB. NCs with a size of about 100 nm have an excellent photothermal ability, originating from the GRQDs. In addition, the NCs had an interesting redox reaction to glutathione from disulfide bridges (created by an amino functionalized MSNs amidation cystine), and thus, the loaded drugs could be released in a controllable manner [138]. Cancer cells excrete lactic acid, which leads to low pH values in cancerous environments. The covering of GO by wedelolactone (Wed) and indocyanine green (ICG) by π-π interaction has been shown to result in the generation of an ICG-Wed-GO multifunctional anticancer DDS for controlling trimodal synergistic therapy consisting of chemotherapeutic therapy, PTT, and PDT. Wed is a polyphenolic compound, isolated from *Wedelia calandulaceae* or *Eclipta prostrata*, having broad anti-tumor effects through the binding of dsDNA, inhibiting topoisomerase IIα, and blocking DNA synthesis. ICG-Wed-GO NC effectively absorbs near infrared (NIR) laser irradiation and transforms optical energy to heat, generating/ablating reactive oxygen species (ROS), leading to the destruction of tumors. It has been shown that after treatment for 14 days with ICG-Wed-GO and NIR, cancer cells were completely lost in tumor-bearing mice. Furthermore, the insignificant ICG-Wed-GO toxic effect has been established [139]. The ability of GO to change its fluorescence at visible/NIR wavelengths as a function of pH was applied to scan cancerous tissues. For example, Campbell et al. described this type of DDS as a new multifunctional agent for the delivery, imaging, and detection of tumor environments [140]. GO is able to detect the acidic extracellular tumor environments of the HeLa and MCF-7 cells in comparison with non-cancer HEK-293 cells, and possesses excellent characteristics, such as its chemical and optical properties, easy functionalization, large surface area, high thermal stability, suitable conductivity, and larger range of biomedical applications [6]. In addition to detection, GO can protect gene therapeutics from nuclease degradation and streamline treatment by increasing intracellular drug delivery that can be covalently or non-covalently associated with various functionalization approaches [140]. For example, hybrid miRNA provides GO with positive functional groups that could take superiority over targeting and PTT when suppressing cancer cells [27]. MicroRNA-101 (miR-101) is a significant regulatory factor that is downregulated in various cancer types, such as prostate, liver, lung, breast, pancreatic cancer, bladder, and colon cancer. miR-101 is a regulator of autophagy, apoptosis, metastasis, cellular stress, and cancer cell growth. Influencing genes, such as Stathmin1 with miR-101, function as apoptotic accelerators and autophagic suppressors. Peptides, such as cell penetrating peptides, e.g., poly-l-arginine (P-l-Arg), are not only internalized into the cell but also can swiftly transport nanostructures. GO molecules covalently decorated with PEG and P-l-Arg, which are able to reduce the surface of GO and increase NIR absorption by approximately 7.5-fold as compared to that of non-reduced GO, are perspective biomolecules for killing tumor cells by regulating various cell functions, such as proliferation, apoptosis, metastasis, differentiation, autophagy, invasion, and stress. GO-PEG-(P-l-Arg) has a higher payload of miRNA, greater internalization, and facilitates endosomal to fall into the cytoplasm as compared to GO-PEG. In addition, the application of P-l-Arg has been shown to increase the selective transfection of systems in tumors (MCF7, MDA-MB-231) as compared to immortalized breast and fibroblast cells. The application of GO-PEG-(P-l-Arg)/miR-101 and NIR irradiation has been shown to induce a 68% occurrence of apoptosis in cancer cells and suppress the Stathmin1 protein [27,65].

**Figure 11:**
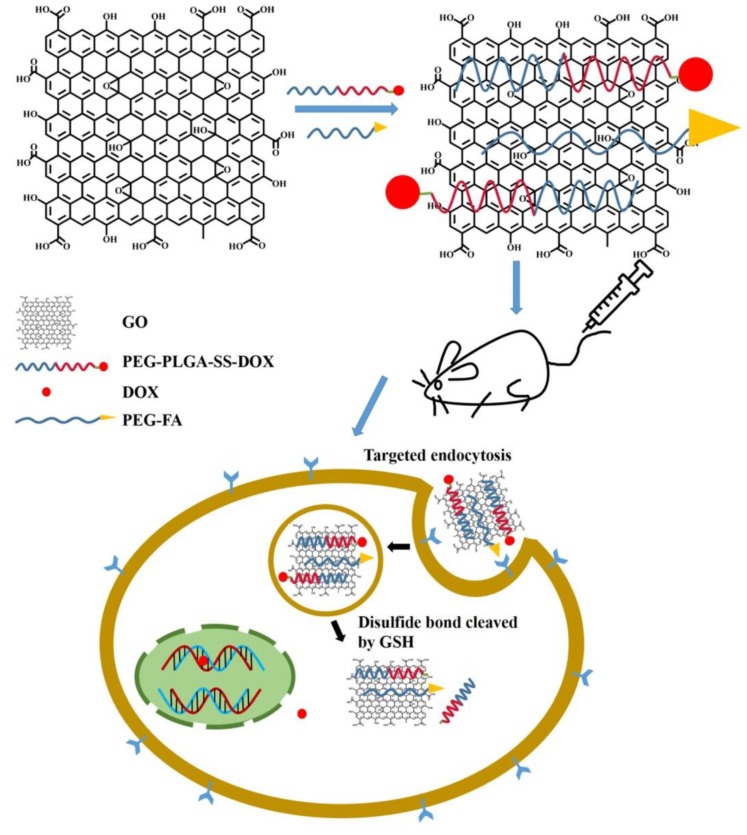
Illustration of active tumor-targeting and sufficient redox-sensitive release of GO/PP-SS-DOX/PEG-FA nanohybrids. Reprinted from [142] with permission from Elsevier, copyright 2019.

Yuan et al. designed CS-coated microspheres, prepared from konjac glucomannan/sodium alginate/GO injected into CaCl_2_ solution with the help of high voltage static electricity, as colon-targeted delivery systems. The performed tests suggest that the CS membrane improved the stability of microspheres and GO ensured a high loading capacity for drugs [141]. On the other hand, other defects can be found that complicate the further use of GO as a drug carrier in the treatment of cancer. Firstly, GO is easily aggregated in electrolyte solutions, e.g., buffered saline. Secondly, drugs could not be effectively released from GO-based DDS due to π-π GO-drug binding (which is usually <40%). Third, untargeted drug release causes adverse effects on healthy cells. Various attempts to solve these problems have been made, for example, to modify GO by PEG to increase the stability of GO. The natural structure of GO destroys covalent bonds. Conversely, non-covalent methods disrupt the internal structure of GO and are therefore more versatile and sensible than covalent methods [142]. Drug release systems can be modulated by stimulus-responsive systems, i.e., specific signals (pH, redox potential, temperature, and light). Redox-responsive systems are such carriers. These include redox-sensitive bonds (e.g., disulfide bond) that are stable in non-tumor tissues/cells but can be cleaved in a redox-triggered microenvironment. Figure 11 represents an active tumor-targeting and redox-sensitive release of DOX based on a complex of GO and PP which is formed by methoxypoly(ethylene glycol) (mPEG) as a hydrophilic part and poly(lactic-co-glycolic acid) (PLGA) as a hydrophobic part. Furthermore, PP was conjugated with DOX through disulfide bonding (SS) and mPEG with folic acid (FA). The resulting complex GO/PP-SS-DOX/PEG-FA showed that it has sufficient dispersibility and that it can serve as a stabilizing agent of GO and an active carrier for a target drug delivery [142].

The covalent interactions of the carboxyl groups of GO with the amine group of peptides or polymer surface of rGO lead to a decrease of the content of oxygen, and, on the other hand, GO conductivity was increased in the biological fluid. Thus, modification techniques such as the application of PEG enhance the dispersity and solubility of GO, even when in salt or serum for a long time [24].

Especially, anticancer drugs, but also drugs from different therapeutic classes, have become a part of GR-based DDSs designed and developed by a number of scientific teams around the word. Multiple examples of different nanoformulations with various therapeutic agents based on GR, GO, and rGO are discussed below.

A DDS based on arginine-glycine-aspartic acid (RGD)-conjugated GRQDs was prepared and used to load DOX. The DOX-RGD-GRQDs showed high pH dependent DOX release. Contrary to free DOX, substantial cytotoxicity to human glioma U251 cells was exhibited by the investigated conjugates within a broad range of DOX concentrations. The results of the cellular uptake of DOX-RGD-GRQDs show that not only DOX, but also some GRQDs penetrated into cell nuclei after 16 h of incubation [143]. Ginsenoside Rh2 (triterpene glycoside saponin), a new anti-tumor natural agent, extracted from ginsenosides, has been shown to inhibit the growth of some types of tumor cells, including pancreatic, breast, gastric, and prostate cancer, as well as hepatocellular and skin squamous cell carcinomas. Rh2-treated GO (GO-Rh2), lysine-treated highly porous GR (GR-Lys), arginine-treated GR (GR-Arg), Rh2-treated GR-Lys (GR-Lys-Rh2), and Rh2–treated GR-Arg (GR-Arg-Rh2) were developed by Zare-Zardini et al. Interestingly, the cytotoxic activity of GR-Arg, GR-Lys, GR-Arg-Rh2, and GR-Lys-Rh2 against cancer cell lines was higher than that towards non-cancer cells (MSCs). GR-Arg and GR-Lys showed fewer undesirable effects in comparison with non-functionalized GR [144].

**Figure 12:**
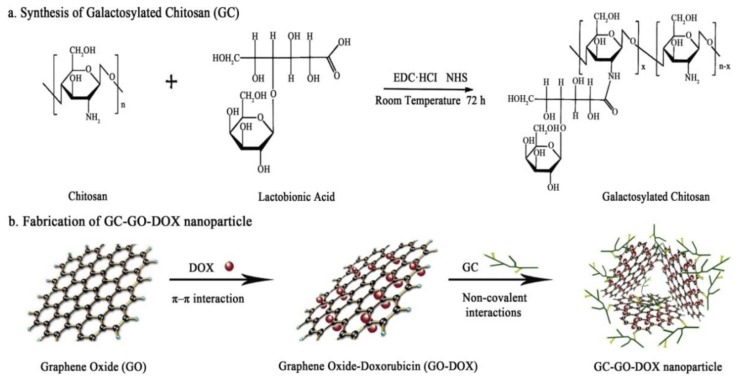
Schematic representation of the synthesis of galactosylated chitosan (CS) and the fabrication of galactosylated-CS/GO/DOX nanoparticles. Reprinted from [145] with permission from Elsevier, copyright 2019.

Galactosylated-CS/GO loaded with DOX has been demonstrated to possess a high drug loading capacity (1.08 mg/mg drug per polymer), the ability of release of the given drug in a low pH environment, and high cytotoxicity for HepG2 and SMMC-7721 cells. Figure 12 shows the preparation of a galactosylated-CS/GO/DOX complex. [145].

New hydrogel NC films with anti-tumor effects were designed by incorporating GQDs into a carboxymethylcellulose hydrogel and using DOX as a model drug. The investigation of the DDS revealed improvements in swelling in vitro, degradation, water vapor release, and the delivery of pH sensitive drugs, along with insignificant toxicity towards blood tumor cells K562 [146]. Javanbakht et al. prepared a carboxymethylcellulose/zinc-based metal-organic framework/GO NC loaded with DOX, which demonstrated significant cytotoxicity to K562 cells and higher water solubility [147].

Using l-plenylalanine (Phe), β-cyclodextrin (β-CD) was grafted onto GO to improve its stability, drug loading capacity, and biocompatibility. The loading efficiency of the GO-Phe-CD nanocarrier was 78.7%, while the capacity of DOX was 85.2%, and the DOX release rate in the acidic environment of the cancer cells was high. Also, GO-Phe-CD was not toxic and GO-Phe-CD with DOX effectively killed MCF-7 cells [148]. Hydrophilic nanospheres prepared from hydroxypropyl-β-CD (HP-β-CD) and carboxylated GO (GO-COOH) were components of a novel GO-COO-HP-β-CD NC, suitable for use as a DDS for lipophilic drugs with limited water solubility. The produced NC has been shown to possess a high dexamethasone (DEX) loading capacity and degree of water solubility. In vitro cytotoxicity tests proved that the nanospheres were not toxic and are suitable for intravenous use with suitable blood compatibility. It was observed that the biological effects of DEX were not influenced by incorporation into the NC [149].

Electroconductive polymeric films, comprised of hyaluronic acid, gelatin, and poly(ethylene oxide) reinforced by rGO, were utilized in drug release studies to evaluate the suitability of the films as drug carriers. These types of conductive biopolymers possess great potential for carrying drugs for cardiac treatment and regeneration with the purpose to increase conductivity aberrations caused by coronary heart disease and conduction in the heart block. The cardiovascular drug irbesartan was loaded onto the polymeric films, providing the controlled and sustained release of the drug [150].

The application of Ca^+2^ and Ba^+2^ ions as crosslinking agents, CaCO_3_ particles as solid porogen, rivastigmine (RIV, used for the therapy of Alzheimer’s disease), water-soluble tragacanth gum (GT) and GO nanosheets allowed the production of spherical pH-sensitive porous hydrogel beads. The swelling behavior of the beads was influenced by the cross-linker content, cross-linker type, composition of beads, and pH. The in vitro release behavior of RIV from hydrogel beads was found to be significantly pH-sensitive. The control of RIV release can be made via pH changes. The release accelerates when increasing pH from 1.2 (<45% RIV released) to 7.4 (97% RIV released). This is due to ionization of COOH groups in GO and GT at a high pH, which increases the swelling of the hydrogel beads. Cytotoxicity analysis has shown that 98% of cells survive at a bead concentration <125 µg/mL. The GO in the hydrogel caused an increase in swelling and capture efficiency and ensured the controlled release of the entrapped drug [151].

**Figure 13:**
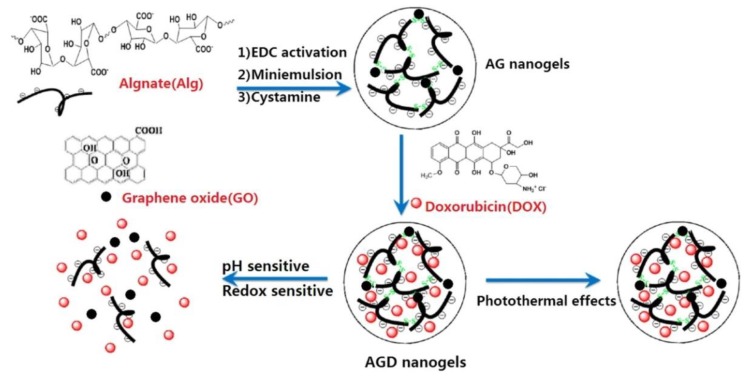
Scheme of synthesis and characterization of the GO-alginate nanogel (AG nanogel) complex with doxorubicin (DOX). The process includes the fabrication of GO-alginate nanogels (AGDs) and their therapeutic effects. Reprinted from [152] with permission from Elsevier, copyright 2019.

A GO-alginate nanogel (AGD) was prepared in situ by incorporating DOX-loaded GO under acidic as well as reducible conditions, simulating a extracellular tumor microenvironment and intracellular components. The internalization of DOX and its long-term accumulation in A549 cells were both improved by the nanogel application. In addition, the nanogel showed photothermal activity against cancer cells. The preparation of AGD is shown in Figure 13 [152]. A GO modified by protamine sulfate and a sodium alginate NC loaded with DOX was proven to have high water dispersibility, uptake by MCF-7 cells, and cytotoxicity towards MCF-7 cells [153].

A dual-sensitivity DOX-based DDS, the surface of which was coated with disulfide bond proapoptotic peptides (KLAKLAK)2 (KLA), was prepared with GO loaded via π-π bonding. The GO carrier was coated by bovine serum albumin (BSA) and the DOX@GO-SS-KLA/BSA DDS, which was stable in biological media, was prepared. The results showed that the reduction and pH inside the cells, respectively, caused the release of KLA and DOX, and synergy was achieved [154].

**Figure 14:**
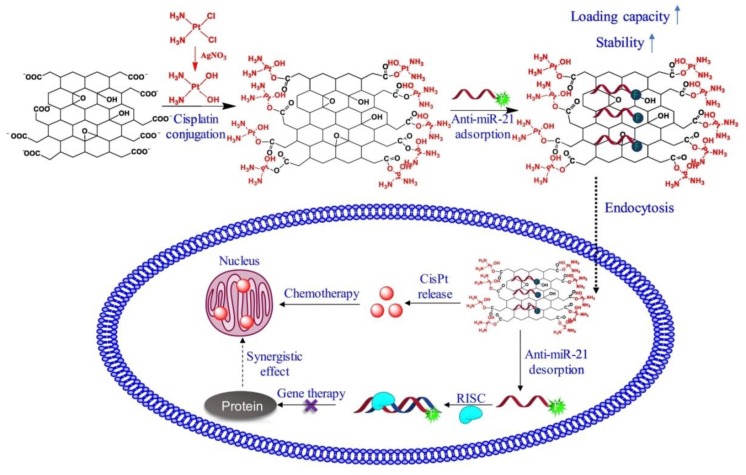
Scheme of the preparation of graphene oxide and cisplatin (CisPt) complex, with the adsorption of Anti-miR-21 for cancer treatment. Reprinted from [115] with permission from Elsevier, copyright 2019.

Cisplatin (CisPt), a strong alkylating antitumor drug, was loaded to GO. CisPt-GO NPs, co-loaded with an antisense microRNA-21 (Anti-miR-21), were tested as a potential RNAi agent. This NC delivered anti-miR-21 and CisPt into A549 tumor cells, which led to strongly improved cell apoptosis and therapeutic efficiency (Figure 14) [115].

PEGylated GO, loaded with curcumin, as an anticancer drug, proved to be an excellent controlled-release DDS for the delivery of curcumin [26]. The conjugation of PEG-PLGA, GO, and folic acid (FA) with DOX, via a disulfide bond, provids a NC with enhanced stability under physiological conditions and DOX liberation in the reductive environment of cancer cells. Targeting nanohybrids showed considerable higher cytotoxicity in vitro than non-targeting nanohybrids. A considerable in vivo antitumor effect was observed, with nearly no systemic toxicity in B16 tumor-bearing mice [137].

Alendronate-functionalized GO nanosheets (DOX@PEG-NGO-ALs) enhanced the deposition of DOX in the metastasis of bones. The results indicate that the PEG-NGO-ALs DDS could be used for the treatment of bone related disorders, e.g., osteoporosis and Paget’s disease [155].

Functionalized phthalic anhydride, with CS followed by 4-cyano, 4-[(phenylcarbothioyl) sulfanyl] pentanoic acid as a chain transfer agent (CTA), to give a CS-CTA macroinitiator, was copolymerized with a methacrylic acid monomer (MAA) to obtain a polyacrylic CS graft (methacrylic acid copolymer) (CS-g-PMAA). This GO was used for the preparation of a copolymer, and the CS-g-PMAA/GO NC was loaded with DOX. The experimental results confirmed the excellent biological and physicochemical properties of the developed NC, which can be applied as a DDS for cancer chemotherapy [156]. A stimuli-responsive GO/polymer brush NC (GCANBNs), synthesized via the polymerization of acrylated β-CD, acrylic acid, and *N*-isopfropylacrylamide from the GO surface, was loaded with both DOX and methotrexate (MTX). Drug release observations demonstrated reasonable NC performance with respect to temperature and pH sensitivity. In addition, the MTT study showed that filling improved drug efficacy [157]. The functionalization of the GO surface with aminated triphenylamine (A-TPA) and 1,3,5-benzenetricarbonyl trichloride (BT) led to the revitalization of its fluorescence properties, and the NC was studied as a new fluorescent DDS. The toxicity of GO-A-TPA-BT NC on human breast cancer cells T47D was investigated. The DDS was loaded with DOX, and the drug release profile indicated that, at an acidic pH level, further release occurs, due to smaller interactions between DOX and the hybrid [158]. A significant in vitro cytotoxic effect on breast cancer cells MDA-MB-231 was shown by GO-methyl acrylate (MA) NPs coated with FA and loaded with paclitaxel (PTX). Moreover, in vivo (in DMBA induced breast cancer rats) studies were performed and demonstrated that treatment with this nanosystem regulates the levels of mitochondrial citric acid enzymes to be near normal [159]. Two-dimensional CS polymerized GO and PVP polymerized GO NPs decorated with FA and loaded with camptothecin (CPT) were prepared by Deb and Vimala. The study indicated that CS-polymerized NPs were more suitable for biomedical applications in terms of their hemolysis, anti-inflammatory properties, and cellular toxicity against MCF-7 cell lines compared to PVP [160]. GO functionalized with hydrophilic and biodegradable PVP or β-CD was loaded with the water-soluble aromatic anticancer drug SN-38 (7-ethyl-10-hydroxy camptothecin). The release of SN-38 loaded into both GO-PVP and GO-β-CD via π-π bonding was tested in different media at pH 7 (water, neutral medium), pH 5 (acidic buffer), and pH 12 (basic buffer). The carrying capacity and cytotoxic activity of SN-38 loaded on the functionalized GO were both tested, and it was found that the cytotoxicity of SN-38–GO–PVP against the human breast cancer cell MCF-7 was comparatively higher than that of the free SN-38 and GO-β-CD [48]. Gefitinib and quercetin, loaded in combination into PVP-functionalized GO NPs, tested in PA-1 ovarian cancer cells, were significantly more toxic than the systems loaded with the individual drugs and the free drugs, and their effects were compared to those on IOSE-364 ovarian epithelial cells. The combined system afforded a stronger effect of the combinatorial approach and a higher effectivity of chemotherapeutic delivery [25].

The treatment of solid prostate tumors by intratumorally-injected GO covered by PdNP (GO-PdNPs) was found to be more effective in a prostate cancer PC3 xenograft mouse model (PC3 cells) than GO or PdNPs alone. Apoptosis caused by the synergy of the photothermal effect and generation of ROS was considerably enhanced by the NIR irradiation of the treated cells. In addition, the insignificant toxicity on organs of GO-PdNPs was an advantage here [161]. GO functionalized with FA and polyethyleneimine (PEI) provided FA-PEI-GO, which was intended to carry two new copper (II) complexes into the folate-receptor-positive nasopharyngeal carcinoma cells HNE-1 and CNE-2. The cytotoxicity of the complexes showed that the IC_50_ values towards the two cell lines were 17.7 ± 1.2, 13.2 ± 1.9 and 6.7 ± 0.8, 2.9 ± 0.7 μM, respectively [162]. Alumina (AlO(OH)) induces antibody-mediated immunity, but due to poor stimulation of cell-mediated immunity, it is not suitable for tumor immunotherapy alone. However, it is important to note that AlO(OH)-modified GO nanosheets (GO-AlO(OH)) can stimulate the cellular immune response [163].

**Figure 15:**
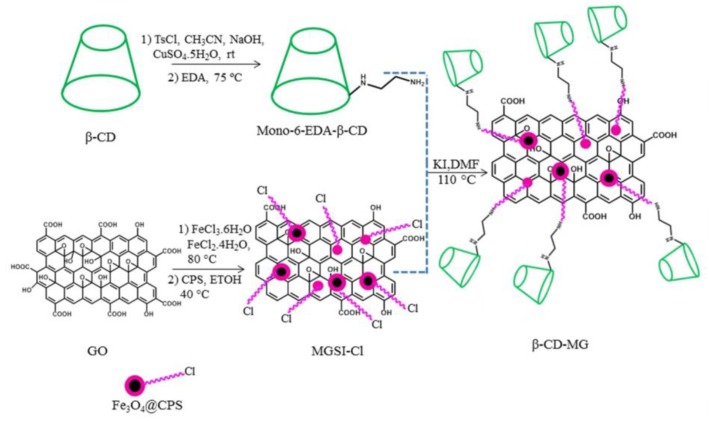
Synthesis of β-CD-MGO. Reprinted from [164] with permission from Elsevier, copyright 2019.

The synthesis of a MGO as DDS is illustrated in Figure 15. A β-CD grafted MGO NC (β-CD-MGO) was investigated for use as a DDS for DOX and MTX. The obtained drug loading efficiencies of the DDS were approximately 37.4% and 23.4% for DOX and MTX, respectively. It was determined that the NC has a better drug release behavior in cancer cells conditions than in non-cancer cell conditions. The screening of toxicity showed that the NC, where cell viability is higher than 80%, is nontoxic for K562 [164].

A CS/NC sodium alginate functionalized magnetic iron oxide GO (MGO-CS/SA) NC loaded with DOX had a diameter of 0.5 μm, a thickness of 40–60 nm, and a drug content of 137% w/w. The system had good solubility and the drug release was pH dependent. In vitro studies have shown the possibility of magnetic targeting and the significant photothermal effect of the carrier. Here, system toxicity was concentration-dependent [165]. PEGylated, CS-encapsulated, DOX-loaded oleylamine-modified Fe_3_O_4_ (DOX/OA-Fe_3_O_4_@CS-PEG) NPs, as cancer-specific theranostics for targeted DOX delivery and magnetic resonance (MR) imaging, designed by Xie et al., had a high drug loading capacity of 24.3% and a saturation magnetization of 4.11 emu/g. The results of the in vitro evaluation showed that the empty NC was cytocompatible, while DOX/OA-Fe_3_O_4_@CS-PEG increased penetration into the nuclei of HepG2 and showed higher efficacy in comparison to free DOX [166]. The NC for GRQD and Fe_3_O_4_, conjugated with concanavalin A, a lectin protein, provided GRQD-ConA@Fe_3_O_4_. This multifunctional NC is able to detect HeLa cells with a dynamic linear range of 5 × 10^2^ to 1 × 10^5^ cells/mL, with a detection limit of 273 cell/mL. GQD-ConA@Fe3O4 is also suitable as a nanocarrier for DOX. In the presence of a magnetic field, the DOX concentration in HeLa cells was more than double. In addition, HeLa cells were 13% more sensitive to DOX in this system than normal cells, confirming the selective role of concanavalin A [167]. The in vitro screening of multifunctional hydrophilic magnetic NPs based on egg yolk-functionalized Pluronic™ F-127 (GYSMNP@PF127), with a particle size of 180 nm, a negative surface charge, and high hemocompatibility, showed a high capacity of 91% w/w for DOX, with a high heating effect with an alternating (AC) magnetic field (internal energy loss in the range of 2.1 to 2.7 nHm^2^/kg), a controlled release of the pH responsive drug, and thermal stimulus (46% at the acidic pH of the tumor and 7% at the physiological pH) when in an alternating magnetic field (MF) [168]. The multifunctional NPs, with a diameter of 71.8 nm and zeta potential of −333.07 ± 0.07 mV, prepared from GO coated with superparamagnetic iron oxide nanoparticles (SPIONs) and poly(lactic-co-glycolic acid) (PLGA), was able to load 3.04 ± 0.46% radiosensitizing 5-iodine-2-deoxyuridine (IUdR) and provided targeted delivery to gliomas. The saturation magnetization was 15.98 emu/g. Treatment with IUdR/GO/SPION/PLGA and external magnetic field caused a reduction in cancer volume in rats compared to buffered saline, IudR, and SPION/IUdR/NGO/PLGA-treated rats [169].

CPT loaded to doubly decorated MSNs grafted with fluorescent conjugates, coated with polydopamine (PDA) and GO layers, was investigated by Tran et al., in relation to targeted delivery and controlled release. These multifunctional NPs demonstrated low cytotoxicity, but high cytotoxicity against SH-SY5Y cells. GO-wrapped MSNs showed effective drug delivery, as GO wrapping enhanced their photothermal heating effect, efficient endocytosis into cells, and dispensability in water solutions. In addition, anti-human epidermal growth factor receptor-conjugated MSNs demonstrated a high specificity, which led to more enhanced anti-tumor effects in vitro [170]. Biomaterials based on various silicate derivatives are important as drug carriers. The in vitro antibacterial efficacy of GO-SiO_2_ and rGO-SiO_2_ NCs against *E. coli* and *S. aureus* was observed, and rGO-SiO_2_ demonstrated a higher antibacterial effect against both bacterial strains than GO-SiO_2_ NC [171].

A novel multifunctional fluorinated GO (FGO) nanocarrier surface, modified with oxygen groups, and functionalized with FA pre-linked amino-polyethylene glycol (PEG), provided suitable solubility and good targetability against tumor cells with FA receptors. Furthermore, FGO-PEG-FA can be loaded with DOX and CPT either individually or together in a controlled manner. FGO also showed photoluminescence and strong NIR absorbance. Therefore, these types of DDSs can additionally be used as an effective PTT system. Based on these facts, a three-in-one therapy model was developed, combining mixed drug treating and PTT. A synergistic chemo-photothermal therapy agent was also found in case of PDA-doped mesoporous silica-coated reduced GO (rGO/MSN/PDA) loaded with DOX. This NC showed high therapeutic effects against hepatocellular carcinoma cells [172].

Recently, GR-based NCs have had much attention paid to them in the context of anti-tumor therapy, due to their rare physicochemical characteristics. The combination of CPT-loaded rGO-AgNPs, with a mean size of 10 nm, uniformly distributed on GR sheets, was evaluated against HeLa cells. rGO-AgNPs strongly potentiated CPT-induced cytotoxicity, apoptosis, and autophagy in the HeLa cell line. In effect, this NC can be used for the treatment of cervical tumors, with strong synergism with CPT or any other antitumor agents [173].

## 9. Conclusions

Nanotechnology is regarded as one of the key technologies of the 21st century. Nanoscale materials change their physical and chemical properties, and thus, old materials are commonly innovated and used practically in all fields of human activity. Graphene is just one of these innovative nanomaterials that been intensively investigated, similarly to other graphene-based nanomaterials. These materials can be widely modified, and therefore, nanomaterials based on graphene can be applied as ultrasensitive sensors, advanced catalysts, superadsorbents, and nanocomposites. They are used in optoelectronics/electronics, chemical solar cells, fuel cells, supercapacitors, rapid charging/discharging batteries, etc. In addition, these materials, depending on the relevant modification/substitution, exhibit a broad spectrum of biological effects, ranging from antiproliferative (to help to suppress the growth of microbial pathogens or cancer cells) effects, to materials suitable for tissue engineering and regenerative medicine applications (e.g., to support stem cell growth and the proliferation and differentiation of cells). Moreover, they have been widely investigated as delivery systems for the controlled release and/or targeted delivery of drugs/agrochemicals in biomedical, pharmaceutical, and agricultural applications. However, it should be noticed that graphene-based nanomaterials can be considered as specific non-biodegradable/non-metabolizable materials that are able to generate various adverse cellular/tissue effects, depending on the specific size of the nanomaterial and surface modification, and, therefore, reactivity. They can be deposited in the human body or environment, thus bringing potential environmental, health, and safety risks. Despite the aforementioned significant benefits of nanomaterials in biomedical applications (e.g., drug delivery carriers), an increased level of attention should be devoted to the potential “intrinsic” toxicity of these nanomedicines caused by particle size, which is able, within side effects, to induce various pathological processes which can result in various adverse/hazardous effects in animals and humans. Therefore, many materials are not suitable due to their aforementioned toxicity, but some graphene-based materials have demonstrated many benefits at acceptable toxicity levels, and thus appear to be promising areas for further development. However, it will take a long time for any of these nanosystems to enter the market for biomedical applications. In addition, all these innovative nanomaterials are highly efficient in a number of non-biomedical applications, however, for their safe use, they must also have high stability to avoid eventual additional environmental pollution.
